# Crosstalk between gut microbiota and gut resident macrophages in inflammatory bowel disease

**DOI:** 10.2478/jtim-2023-0123

**Published:** 2023-12-20

**Authors:** Haohao Zhang, Xueying Wang, Jing Zhang, Yixuan He, Xiumin Yang, Yongzhan Nie, Lijuan Sun

**Affiliations:** Key Laboratory of Resource Biology and Biotechnology in Western China, Ministry of Education. School of Medicine, Northwest University, Xi'an, Shaaxi Province, China; State Key Laboratory of Cancer Biology, National Clinical Research Center for Digestive Diseases and Xijing Hospital of Digestive Diseases, The Fourth Military Medical University, Xi'an, Shaaxi Province, China; State Key Laboratory of Targeting Oncology, National Center for International Re-search of Bio-targeting Theranostics, Guangxi Key Laboratory of Bio-targeting Theranostics, Collaborative Innovation Center for Targeting Tumor Diagnosis and Therapy, Guangxi Talent Highland of Bio-targeting Theranostics, Guangxi Medical University, Nanning, Guangxi Zhuang Autonomous Region, China

**Keywords:** gut microbiota, macrophage, inflammatory bowel disease, bacterial metabolite, in-nate immunity

## Abstract

Macrophages residing in the gut maintain gut homeostasis by orchestrating patho-gens and innocuous antigens. A disturbance in macrophages leads to gut inflamma-tion, causing conditions such as inflammatory bowel disease (IBD). Macrophages ex-hibit remarkable plasticity, as they are sensitive to various signals in the tissue micro-environment. During the recent decades, gut microbiota has been highlighted refer-ring to their critical roles in immunity response. Microbiome-derived metabolites and products can interact with macrophages to participate in the progression of IBD. In this review, we describe recent findings in this field and provide an overview of the current understanding of microbiota-macrophages interactions in IBD, which may lead to the development of new targets and treatment options for patients with IBD.

Macrophages are an essential population of tissue-specific innate immune cells that function as “housekeepers”. These cells are widely distributed in most tissues, including the intestine, liver (Kupffer cells), bone (osteoclasts), alveola (dust macrophages) and central nervous system (microglia). An overwhelming body of literature supports that these macrophages exert crucial roles in inflammation,^[1]^ autoimmune diseases,^[2]^ metabolic diseases,^[3]^ cancers,^[4,]^ and nervous system diseases.^[5]^

The gastrointestinal tract orchestrates the antigens from diet and the gut bacteria. The resident gut macrophages are usually considered as the major gatekeepers, as they play key roles in engulfing pathogens, triggering inflammation, and regulating wound healing. Over the past several decades, intestinal macrophages were believed to be derived only from monocytes. However, with the development of fate-mapping techniques and single-cell sequencing, several subsets of self-maintaining macrophages were recently demonstrated to derive from multiple sources, harbor specific functions and be located within specific niches, suggesting that macrophages have widespread impacts on our health.^[6]^

Macrophages exhibit remarkable plasticity and are sensitive to specific changes in tissues and the cell microenvironment, as well as to a large number of activating molecules. In the past few decades, macrophages are generally believed to directly polarize towards a pro-inflammatory phenotype (M1) or a pro-resolving phenotype (M2). These phenotypes have distinct cell surface markers and biological characteristics, and they produce specific cytokines. When monocytes are activated by Toll-like receptor 4 and nuclear factor-κB, they acquire an M1 phenotype, and subsequently produce tumor necrosis factor (TNF), interleukin (IL)- 1β, IL-6, IL-12, IL-23, and CC motif chemokine ligand 2 (CCL2). In contrast, when activated by glucocorticoids, IL-4, IL-13, and IL-10, the cells acquire an M2 phenotype and polarize into different subtypes (M2a, M2b, M2c, and M2d), thereby producing distinct cytokines according to the highly heterogeneous tissue microenvironment signals.^[7]^

However, from the current point of view, the tissue microenvironment provides multiple signals directing the monocytes towards a much more complicated macrophage phenotype. Accordingly, the classic M1/M2 polarization cannot explain this regulation. In the gut, macrophage development and maintenance are heavily dependent on the environmental signals, like colony stimulating factor (CSF) 1, CSF2, IL-34, CX3CL1, transforming growth factor-β, and IL-10.^[7]^ During recent years, the gut microbiota has been highlighted for their critical roles in macrophages diversity and function. Microbiome-derived metabolites and products can interact with macrophages and participate in the progression of inflammatory bowel disease (IBD). Here, we firstly describe the heterogeneous macrophages within the intestinal tissue and their roles in normal physiology states and IBD. How environmental signals induce macrophages diversity and polarization, particularly the gut microbiota, and how their interactions exert their protective or harmful activities in health and inflammation, are also discussed. Finally, efforts aimed at developing novel therapies targeting macrophages (mainly by gut microbiotatarget therapeutic approaches) are reviewed.

## Functional heterogeneity of niche-specific macrophages in the gut

Over the past few decades, intestinal macrophages were regarded to be derived from blood monocytes replenished by bone marrow progenitors. Although macrophages are seeded by embryonic precursors during the embryo stage, they do not persist into adulthood, and continue to be replenished by bone marrow-derived monocytes after weaning. As the intestine is constantly exposed to foreign antigens, commensal bacteria, and dietary nutrients, appropriate responses to these factors are necessary to maintain tissue homoeostasis. Accordingly, macrophages form the first line of defense against pathogens and contribute to maintaining tissue homeostasis and wound healing.

During the last few years, emerging study models allowed further understanding of the characteristic of the niche-specific macrophages in the gut. The models used Cre recombinase to drive a fluorescent protein expressed in a specific cell lineage, which enable us to understand the developmental origins of various immune cells, including the macrophages. Furthermore, another model, which marks the cells by inducible fate-mapping mouse, makes our understandings in this field more precise, which are reviewed in details in other review.^[8]^ With the development of single cell RNA sequencing (scRNA-seq) technologies, profiling individual cells is now possible *via* next-generation sequencing, then providing an unbiased information about immune cell diversity.^[9]^ Collectively, fate-mapping in mice and scRNA-seq, together with multiphoton imaging and multiparameter flow cytometry, can define novel cell populations and their functions. This is also the case in the discovery of macrophages subgroups.

**Figure 1:**
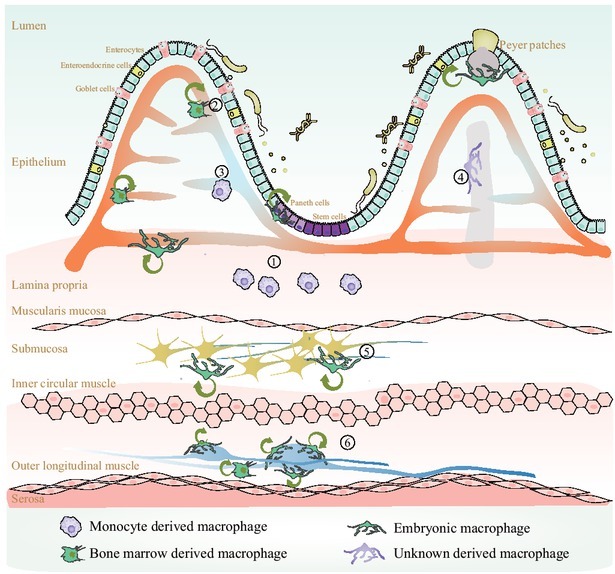
Heterogeneity of gut-specific macrophages. ① Lamina propria macrophages. This subset contains the most abundant macrophages within the gastrointestinal (GI) tract and is constantly replenished by blood monocytes. These cells play critical roles in capturing and eliminating pathogens, engulfing apoptotic cells, regulating oral tolerance, presenting antigens, and improving wound healing. ② Blood vessel-associated macrophages. This subset is bone marrow-derived or embryonic, has a self-maintaining ability, and plays roles in preserving vascular integrity and preventing microbe dissemination. ③ Other blood vessel-associated macrophages. This subset preserves vascular integrity and prevent microbe dissemination. However, this subset is constantly replenished by blood monocytes, as these cells are located very ④ close to the lumen. Lacteal-related macrophages. A subset surrounding the lacteal to capture apoptotic cells and present antigens. ⑤ Submucosal neuron-related macrophages. A subset close to submucous plexus and are self-maintaining. They are involved in supporting neuron survival and regulating GI motility. ⑥ Muscularis neuron-related macrophages. A subset close to the enteric ganglion involved in supporting neuron survival and regulating GI motility.

Majority of the gut macrophages originate from the bone marrow-derived macrophages and are located within the villi and surrounding the mucosal vascular network. On the other hand, there is a small number of gut macrophages derived from embryonic progenitors, and these macrophages can self-maintain and persist into adulthood. Furthermore, they colonize specific niches surrounding the enteric neurons and vasculature in the submucosa. Due to their high plasticity, macrophages express conventional and tissue-specific surface markers. Based on surface marker expression, gut macrophages can adapt to their environment and more conducive to their various function. In this section, we focus on the recent advances in understanding the diversity of macrophages subsets and their specialized functions in the intestinal tissue (Figure 1).

### Macrophages in the lamina propria

The intestinal epithelium is continuously exposed to antigens. To protect the host against these antigens, macrophages are spread across the gastrointestinal tract, particularly in the lamina propria. However, they are short-lived because of they are continuously exposed to poisonous agents from the gut lumen; they are constantly renewed by Ly6C^hi^ monocytes in circulation *via* the CCL2-CCR2 axis. Monocytes are converted into macrophages in the lamina propria, a process known as the “waterfall” of monocytes.^[10]^ During this process, the Ly6C^hi^ monocytes recruited from the blood acquire major histocompatibility complex type II (MHCII), thus the Ly6C expression is lost. Subsequently, expression of F4/80, CD64, and CX^3^CR1 are upregulated. This process gives monocytes their mature macrophages phenotype characterized by significantly different gene expression from monocytes, including elevated anti-inflammatory cytokines IL-10 and lower pro-inflammatory cytokines (IL-6, iNOS, *etc*.), expressing genes involved in phagocytosis and the complement pathway, acquiring scavenger receptors, and ultimately gaining the ability of unresponsiveness to Toll-like receptor ligation.

Since this macrophages subgroup resides directly underneath the epithelium, it can capture and eliminate bacteria that invade the tissue, and it can support the gut barrier. Consistent with these roles, macrophages display high expression of genes related to phagocytosis, including Merk, Cd206, Axl, CD36, and Itga5. ^[11]^ Despite the fact that macrophages express amount of pattern recognition receptors on their surface, including Nod-like, Toll-like, RIG-I-like, and C-type lectin receptors, macrophages generally remain unresponsive and do not release pro-inflammatory cytokines after the ingestion of harmless bacteria or food antigens to prevent the influx of other immune cells and maintain tissue homeostasis. ^[12]^

Macrophages in the lamina propria also play critical roles in oral tolerance by participating in the presentation of food antigens. Similar to dendritic (DC) cells, macrophages send protrusions between enterocytes and acquire antigen from the lumen without impairing epithelia integrity. In the intestinal lumen, soluble antigens from the diet are efficiently taken up byCX3CR1^+^macrophages, which are dependent on the CX3CL1-CX3R1 axis. As soon as CX3CR1^+^macrophages receive antigens, they transfer peptide-MHCII complexes *via* connexin-43 dependent gap junctions to CD103^+^ DCs, then migrate to the mesenteric lymph nodes and present antigen to naïve T cells.^[13]^ Additionally, lamina propria macrophages can also produce IL-10, leading to the expansion of Foxp3^+^ regulatory T cells, which also aids in oral tolerance and gut homeostasis.

Furthermore, it has been shown that macrophages promote mucosal repair. Lamina propria macrophages have been proven to activate the Wnt signaling pathway in the epithelium layer, thereby promoting epithelial regeneration. CSF1-dependent macrophages have been reported to be essential in maintaining the intestinal stem cell niche in the small intestine. CSF1 deficiency results in impaired epithelial differentiation and renewal, suggesting the critical role of macrophages.^[14]^ Similarly, IL-6, IL-8, IFNγ, and TGF-β1 released by macrophages contribute to the maturation of the intestinal epithelium. Phagocytosis of macrophages also play important roles in wound healing and epithelial integrity. Following dextran sulfate sodium administration, macrophages exhibit significant changes, including the removal of CX3CR1^int^macrophages and restoration of CX3CR1^hi^macrophages. ^[14]^ Macrophages are also important during inflammation resolution. Their phagocytic activity against apoptotic cells, bacteria, and bacterial wall components are potential pathways through which macrophages are involved in wound healing. Moreover, phagocytosis facilitates activation of a transcriptional program that fights inflammation.^[15]^ Macrophages also produce different mediators and cytokines, including prostaglandin E2 and WNT ligands, which are involved in the proliferation of epithelial progenitors and endothelial cell physiology. ^[16]^ In the distal colon, macrophages sense fungal toxins and prevent their absorption by inserting balloon-like protrusions in the epithelium, which largely protects gut barrier integrity.^[17]^

Traditionally, macrophages have an extremely weak ability to present antigens compared to that of DCs; however, given that macrophages express high levels of MHCII, these cells may participate in antigen presentation. A recent study demonstrated that macrophages can cross-present to activate CD8^+^ T lymphocytes to combat tumors and intracellular pathogens.^[18]^ Furthermore, macrophages promote further differentiation of antigen-specific T cells, as well as induce and maintain commensal-specific Th17 cells through IL-1β secretion. Macrophages may also indirectly affect T cells by influencing conventional DC differentiation. Macrophages secrete IL-1β and then enhance the ILC3 to produce CSF2, which can control conventional DC differentiation into lamina propria macrophages. ^[19]^

### Blood vessel-related macrophages

Aside from the classical macrophages in the lamina propria described above, other specialized macrophages subsets surround the blood vessels in the lamina propria. They are self-maintaining and exhibit a remarkably diverse transcriptome compared to macrophages in the lamina propria. They upregulate genes related to angiogenesis, including Tnfaip2, Ecm, Mmp2, Hif1a and Mmp14. This macrophage subgroup is essential for the vascular integrity and prevent microbe dissemination into the vascular system.^[20]^

Interestingly, another macrophages population surrounds the blood vessels in the villi. These mucosal perivascular macrophages in microcirculation play critical roles in protecting the vascular system from bacterial dissemination, similarly to the self-maintaining blood vessel-related macrophages. However, because they are located very close to the lumen and are constantly exposed to bacteria, these cells exhibit high turnover and are largely dependent on circulating CCR2^+^ monocytes.^[21]^

Another subset of CD169^+^macrophagesis located close to the central lacteal and lymphatic vessels at the base of the villi in the small intestine. Although they are identified to be involved in capturing apoptotic cells, immune complexes, viruses, and antigen tolerance, their precise origin remains still elusive.^[22]^

### Submucosal neuron- and muscularis neuron-related macrophages

Beneath the submucosa, the submucous plexus regulates intestinal peristaltic movement, glandular secretions, electrolyte and water transport, and local blood flow. Recently, populations of neuron-related macrophages were found surrounding both the submucosal neuron and muscularis neuron. These subset populations are present at birth and are self-maintaining. In contrast to macrophages in the lamina propria, these cells are far away from the intestinal lumen, gut bacteria, and food antigens, and are critical for neuronal survival and intestinal motility.^[23]^

Macrophages located in this region receive signals from neurons and upregulate genes enriched in the microglia, including Gpr34, Fcrls, Mef2a, and Hexb. They are crucial for survival of the submucous plexus neuron, as loss of this macrophage subgroup leads to neuronal apoptosis and impaired neuron-related anion secretion.^[24]^ In a steady state, this population is largely derived from the embryo and rarely depends on circulating monocytes. However, depletion of these macrophages can induce the recruitment of bone-derived macrophages to this region. Notably, in this region, neuron and macrophages interactions are critical for gut transit. Neurons produce CSF1 to support the maintenance of macrophages, as macrophages were found to be completely absent from CSF1-deficient mice. Additionally, macrophages produce bone morphogenetic protein-2 (BMP2) to support neuronal function, as depletion of macrophages causes caspase-3-related apoptosis and the decreasing over 50% of neurons, accompanied by low intestinal transit and impaired gut peristalsis.^[25]^ A recent study identified the PMP22^+^ muscularis macrophage subgroup among the population. The oligodendrocytes express PMP22, a cell-adhesion molecule, which allows them to interact with enteric neurons.^[26]^

### Other macrophages within the intestine

The scRNA-seq revealed a novel subset of macrophages near Paneth cells in the base of crypt. Their impairment led to a reduction in intestinal stem cells and aberrant differentiation.^[25]^ Paneth cells produce CSF1 and Wnt3 to maintain macrophages and intestinal stem cells, respectively.^[25]^ A distinct population of macrophages around the Peyer patch was also reported. These macrophages exhibit a remarkably different transcription pattern from that of classic macrophages, which is characterized by elevated expression of phagocytosis-, antimicrobial-, and antiviral-related genes. Two macrophages subsets (Tim4^+^ and Tim4^-^ CD4^+^macrophages) have been found in the Peyer patch and function to engulf particles and clear apoptotic cells.^[27]^

## Macrophages in IBD

Recently, there has been a lot of studies have focused mainly on the disturbed adaptive immunity in patients with IBD; however, genome-wide association have highlighted the effects of the innate immune on IBD. Increasing evidence has indicated that some IBD susceptible loci were regulated by the lipopolysaccharide (LPS) or the microenvironmentinduced macrophages differentiation.^[28]^ Similar studies revealed a causal link between altered macrophages differentiation and defects in intestinal inflammation resolution, defective microorganism clearance, and excessive cytokine secretion in patients with IBD.^[29]^

**Figure 2:**
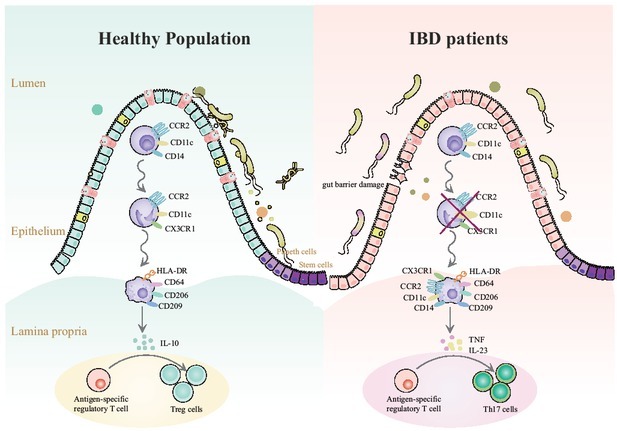
Alteration of lamina propria macrophages in patients with inflammatory bowel disease.

Referring to the roles of gut resident macrophages in IBD, most studies mainly focused on the subtype of macrophages in the lamina propria. Under steady state, the macrophages are constantly replenished by blood CD 14^hi^monocytesand then later progress through a strictly organized series of intermediary cells into IL-10-producing CD11c^-^CCR2^-^ CX3CR1^-^HLA-DR^+^CD206^hi^CD^209hi^mature macrophages. These cells do not secret pro-inflammatory cytokines following exposure to gut bacteria. However, in IBD patients, this process is disrupted, large numbers of CD 14^hi^ monocytes are recruited, and immature macrophages (CD11c^hi^) are largely increased. These macrophages secrete pro-inflammatory cytokines following exposure to commensal bacteria.^[30]^ Notably, in IBD patients, circulating monocytes are characterized by excessive secretion of Il-23 and TNF, which are thought to participate in the early response to pathogens (Figure 2).^[31]^

In healthy populations, human CD14^+^CCR2^+^CD11c^+^ monocytes derived from the blood infiltrate the lamina propria, where they become CCR2^+^CD11c^+^CX3CR1^+^ monocyte-like cells through a series of intermediate cell populations and then become mature macrophages, which secrete IL-10 to improve regulatory T cell expansion. However, in IBD patients, the altered microenvironment in the gut tissue leads to dysregulation of this process. The blood-derived monocytes display CX3CR1^+^, CCR2^+^, CD11c^+^, CD14, CD64, CD206, and CD209. Immature Macrophages induce excessive TNF and IL-23 secretion, and then increase Th17 expansion.

## Gut microbiota and gut resident macrophages interaction in IBD

As described above, monocyte recruitment, macrophages infiltration, and macrophages polarization are sensitive to microenvironmental changes. Trillions of microorganisms colonize the gastrointestinal tract, and their composition and function are related to a large number of diseases, such as autoimmune, inflammatory, metabolic, and nervous system diseases. Although the complicated effects of the gut microbiota are widely documented, immunomodulation by the gut microbiota is emerging as one of the most important pathways by which microbiota exert their roles.

### Gut microbiota and IBD

The gut microbiota composition of IBD patients has been evaluated. The beneficial bacteria, like those involved in short-chain fatty acids (SCFA) production and in tryptophan metabolism, largely decreased in IBD. For example, *Clostridium cluster IV* and *XIV* species, two SCFA-producing bacteria, significantly decreased in patients with IBD.^[32]^ The abundance of several Enterobacteriaceae species decreased, among which is *Faecalibacteriumprausnitzii*, a well-studied beneficial bacterium belonging to phylum Firmicutes and secretes anti-inflammatory metabolites.^[33]^ Overgrowth of intestinal pathogens is another feature of patients with IBD. The IBD patients and mice models exhibit blooms of specific Proteobacteria (*e. g.*, adherent and invasive *Escherichia coli*).^[34]^

More importantly, these changes are related to recurrence. In Crohn’s disease, a low abundance of *F. prausnitziiwas* detected in the ileum, increasing the risk of disease recurrence postoperatively and endoscopically after six months.^[33]^ Tryptophan is the substrate of several *Peptostreptococcusspecies* and can be metabolized to indole acrylic acid derivatives. These metabolites promote mucosal barrier function and reduce inflammatory responses.^[35]^ In another study, changes in *Lachnospiraceae* and *Ruminococcaceae* species were associated with high disease recurrence and poor responses to anti-TNF therapy in Crohn’s disease patients.^[36]^

Whether gut dysbiosis is caused by or resulted from IBD remains unclear; however, it appears to proceed as a two-stage process.^[37]^ In the early stage, gut pathogens accumulate, and susceptible genes may precede the onset of colitis. Two pathogens, *Helicobacter hepaticus* and *Mucispirillumschaedleri*, trigger spontaneous colitis in genetically susceptible mice.^[38,39]^ In the late dysbiosis stage, as a consequence of gut inflammation, higher levels of oxygen in the intestinal lumen, available nitrate, and host-derived electron acceptors drive overgrowth of *Enterobacteriaceae*.^[40]^

### Crosstalk between the gut microbiota and intestinal macrophages

As a key part of the innate immune system, interactions between macrophages and the gut microbiota orchestrate a proper immune response during intestinal inflammation. Macrophages signals are essential in regulating the gut microbiota. Numerous studies have demonstrated that macrophages depletion by clodronate administration led to large alterations in the intestinal bacteria profile, which are characterized as increases in Firmicutes, specifically the *Lactobacillaceae* and *Clostridiaceae* families.^[41]^ In adult zebrafish, irf8 (a key transcription factor involved in macrophages polarization) mutants cause a largely loss of intestinal macrophages and destabilization of the gut microbiota.^[42]^

Additionally, the gut microbiota is indispensable for maintaining the macrophages pool. Csf2 plays a crucial role in myeloid lineage differentiation and macrophages function. In newborns, CSF2 production is absent, but its level increases slightly at day 7 after birth and greatly increases by day 14. This trend is similar to the increasing complexity of the gut microbiota, suggesting that intestinal commensal-driven signals modulate the production of Csf2 and homeostasis of gut resident macrophages.^[19]^ Particularly, in the lamina propria, macrophages are continuously exposed to the microbiota, and inflammation leads to loss of resident macrophages and recruitment of blood monocytes. Compared to conventionally colonized controls, the number of intestinal macrophages in germ-free mice was reduced significantly, and CD11^+^CD206^int^CD121b^+^ and CD11^-^CD206^hi^CD121b^-^ macrophages populations decreased, supporting the correlation between the gut bacterial abundance and intestinal macrophages number.^[43]^ As described above, a special macrophages subset has been detected surrounding the blood vessels within the villi, which are also dependent on blood monocytes. If there was no microbiota, this macrophage subtype largely reduced and the blood vessels were uncovered to enable increased dissemination of pathogens into the circulation.^[22]^ Similarly, after treating mice with antibiotics, muscularis neuron-associated macrophages secreted less BMP2 to support neuron cells; correspondingly, enteric neurons produced less CSF1. This disturbance in crosstalk led to gastrointestinal dysmotility.^[44]^

### Possible pathways gut microbiota affecting intestinal macrophages

**Figure 3:**
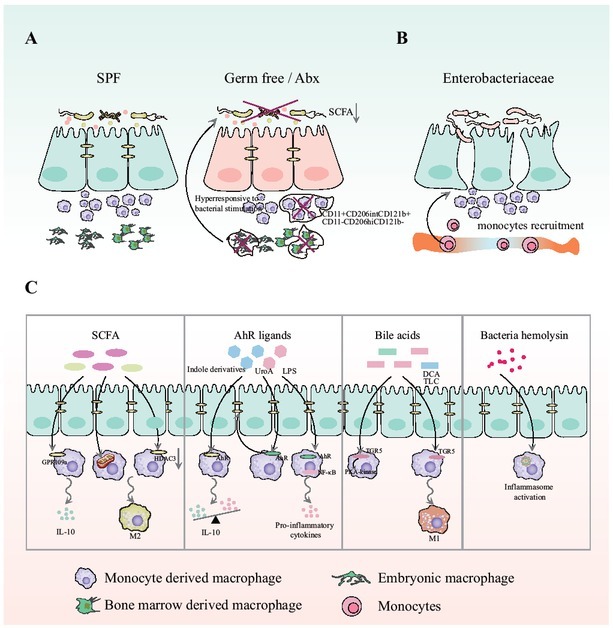
Gut microbiota and intestinal macrophages. A. Under normal conditions or in specific pathogen-free mice, the commensal microbiota is involved in macrophages differentiation and maintenance. However, when the commensal microbiota is depleted by ABX or in GF mice, both monocytederived and long-lived Macrophages are greatly reduced, particularly the CD11c^+^CD206^int^CD121b^+^ and CD11c^-^CD206^hi^CD121b^-^ subgroups; B. Enterobacteriaceae species are responsible for recruiting Ly6hiCCR2^+^ monocytes in the blood. C. Metabolites are the main signals from the gut commensal microbiota. Short-chain fatty acids (SCFAs), indole derivatives, lipopolysaccharide (LPS), and bile acids can act as ligands for G-coupled protein receptor, AhR, or transmembrane G protein-coupled receptor 5 (TGR5), activate these receptors or molecules (HDAC3), exert important regulatory influences on both intestinal macrophages polarization and their function. Bacterial hemolysin is critical for driving persistent activation of inflammation in Macrophages.

How resident bacteria stimulate monocyte recruitment, macrophages polarization, and cytokine release in the gut is poorly understood. However, recent studies have provided insight into these interactions. Several species were reported to be involved in this process. In colitis models induced by dextran sulfate sodium, Enterobacteriaceae species were responsible for recruiting Ly6hiCCR2^+^ monocytes in the blood.^[45]^ Other studies showed that the gut microbiota affects intestinal macrophages directly by releasing metabolites or bacterial components, such as bile acids, aryl hydrocarbon receptor (AhR) ligands, short-chain fatty acids (SCFAs), urolithin A, LPS, and bacteria hemolysin. In this section, we describe how the gut microbiota may affect the intestinal macrophages pool (Figure 3).

### SCFAs

SCFAs are key metabolites derived from colonic microbiota-accessible carbohydrates. In patients with IBD, dysbiosis is typically characterized as a significant reduction in bacteria than can produce butyrate, including *F. prausnitzii* and *Roseburia hominis*, and a reduction in SCFA levels in the feces.^[46]^ Furthermore, SCFAs have important effects on macrophages functions. SCFAs transporter CD98 is strongly expressed in macrophages. Specific deletion of CD98 in CX3CR1^+^macrophagesattenuated DSS-induced colitis, accompanied with a decreased mature macrophage, which indicated that SCFAs are essential for intestinal macrophage maturation.^[47]^ SCFA depletion by antibiotic administration make intestinal macrophages become more hyperresponsive to LPS and produce greatly increased levels of inflammatory cytokines, followed by a long-term increase in the inflammatory Th1 cell response.^[48]^ Accordingly, SCFA supplementation alters the metabolic phenotypes of intestinal macrophages, increases oxidative phosphorylation, and promotes M2 macrophages activation.

Other studies explored the potential mechanisms underlying these observations. Butyrate and niacin can induce IL-10 release by activating the GPR109a receptor in gut resident macrophages.^[49]^ Additionally, butyrate inhibits histone deacetylase 3 and imprints antimicrobial programs in intestinal macrophages.^[50]^ A recent study on macrophages subsets showed that SCFAs were taken up by long-lived Tim4^+^ CD4^+^ colonic macrophages.

### AhR ligands

AhR signaling is crucial for intestinal homeostasis, given its key roles in the immune response, and it affects innate lymphoid cells, macrophages, DCs, neutrophils, intraepithelial lymphocytes, and Th17 cells.^[51]^ A small group of bacteria, such as Lactobacillus, *Peptostreptococcusrussellii*, and *Clostridium sporogenes*, has been reported to produce AhR ligands. Diet-derived indole derivatives and those produced by gut microbes are the major ligands for AhR and include indoleacetic acid, indole acrylic acid, indole-3-acetaldehyde, and indole-3-aldehyde. In macrophages, these derivatives activate AhR pathways, which in turn modulate the susceptibility to intestinal inflammation. Deletion of AhR in CD11^+^DCs and macrophages led to increased susceptibility to dextran sulfate sodium-induced colitis through the addition of specific intestinal epithelial stem cells and differentiation of atypical cells.^[52]^ Additionally, gut microbiota-derived LPS can activate AhR signaling in macrophages associated with a restricted pro-inflammatory response. ^[53,54]^ Similarly, urolithin A, a microbial metabolite, can upregulate epithelial tight junction proteins by acting on bone marrow-derived macrophages *via* activating the AhR pathway and reducing LPS-induced nuclear factor-κB activation.^[55]^

### Bile acids

Bile acids (BAs) are substrates for many bacterial enzymes. Bacteria cause deconjugation, hydrolysis of primary BAs, and conversion of primary BAs to secondary BAs. This conversion function is performed by 7α/β-dehydroxylation enzymes found in a narrow range of clostridial species. Serum and feces samples from patients with IBD show altered BA profiles, including addition of fecal-conjugated primary BAs and reductions in secondary BAs.^[56]^ This may be partly because of gut dysbiosis in patients with IBD.

BAs also play important roles in regulating macrophages. The taurolithocholic acid affects the transcriptome of the macrophages after being treated with or without LPS, including inhibiting the expression of molecules related to phagocytosis, pathogen killing, and immune cell recruitment.^[57]^ Deoxycholic acid has been reported to promote the polarization of macrophages to a pro-inflammatory phenotype.^[58]^ BAs exert their effects by interacting with the transmembrane G protein-coupled receptor 5 (TGR5). TGR5 is a secondary BA receptor expressed on macrophages, and its activation inhibits nuclear factor-κB and decreases the release of peripheral blood monocyte-derived IL-1, IL-6, and TNF, and then reduces liver-resident macrophages response to LPS.^[59]^ Some BAs can induce cAMP activation through TGR5, followed by activation of PKA-kinase and inhibition of cytokine production induced by LPS in macrophages.^[60]^ As BAs are present at high concentrations in the gut, they may also affect Macrophages in the gut.

### Other factors

Other products originated from the bacteria also play critical roles in the function of the macrophages. Hemolysin is a class of toxin produced by gram-positive bacteria. The hemolysin A secreted by Escherichia coli has been identified to activate NLRP3-dependent macrophage cell death.^[61]^

Considering the important roles of macrophages in IBD, interactions between macrophages and the gut microbiota partially contribute to gut inflammation. This condition has been observed in mouse models. Elevation of invaded bacteria in the lamina propria induced macrophages to produce prostaglandins, which then affected epithelium differentiation, reduced goblet cells, and damaged the mucus barrier.^[62]^ Another study utilized an intestinal adherent *Escherichia coli* 541–15toincrease IL-10 producing macrophages, which are helpful for limiting intestinal inflammation and restricting tumor formation.^[63]^

## Therapeutic approaches targeting macrophages involving gut microbiota in IBD

**Figure 4:**
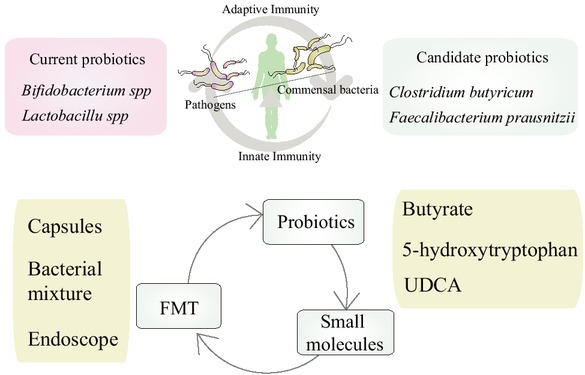
Therapeutic approaches targeting macrophages involving gut microbiota in IBD.

The studies discussed above indicate that the distinct effects of macrophages in gut inflammation may, at least in part, be mediated through the gut microbiota. Considering that the gut microbiota may be suitable for intervention and that current therapies are ineffective in many patients with IBD, novel treatment options are urgently needed. In this section, we review several interventions directly or partly acting through microbiota-macrophages interactions (Figure 4). However, it should be noted that macrophages are not the only immune cells modulated by the gut microbiota, and other immune cells affected by the gut microbiota also participate in resolving gut inflammation.

Probiotics mediates the balance of pathogens and commensal bacteria, then maintains the homeostasis of innate immunity and adaptive immunity. Besides from the classic probiotics (*Bifidobacterium spp.* And *Lactobacillus spp*), other candidate probiotics (*Clostridium butyricum*, *Faecalibacteriumprausnitzii*) may be helpful for clinical remission in IBD; Small molecules, including butyrate, 5-hydroxytryptophan and ursodeoxy cholic acid (UDCA) are also promising candidates for IBD patients; fecal microbiota transplantation (FMT), including bacterial capsules, specific bacterial mixture or bacterial suspension perfusion by endoscope is one of the most excellent pathways to reshape the gut microbiota, which also be a potential therapy for IBD.

### Probiotics

Probiotics have been administered as therapies for several decades. Current probiotics mainly include yeasts, *Bifidobacterium spp.*, and *Lactobacillus spp.* considering their cultivability, aerotolerance, and ability for industrial-scale production. These probiotics can balance the gut microbiota, inhibit pathogen overgrowth, degrade antigens, and stimulate local immunity. A recent meta-analysis of 14 randomized clinical trials explored the effects of probiotics in patients with ulcerative colitis and showed that probiotics induced clinical remission as effectively as 5-aminosalicylic acid.^[64]^ However, the results were not consistent among studies. Thus, whether currently used probiotics can induce remission in patients with IBD remains unclear.

Interestingly, preclinical tests have been conducted to evaluate several other bacteria strains. *Clostridium butyricum*, which can produce butyrate, showed some potential health benefits in a very small study of 17 participants.^[65]^ *F. prausnitzii* has also been regarded as an important candidate for treating patients with IBD.^[65]^ A clinical trial showed that administration of *E. coli* Nissle 1917 had similar efficacy as mesalazinein maintaining remission in ulcerative colitis patients. ^[66]^ Microcins secreted by this strain are antimicrobial and can suppress the growth of Enterobacteriaceae.

### Microbiota-related metabolites

Metabolites are the most important signals from the gut microbiota. A clinical trial study demonstrated that sodium butyrate supplementation may be beneficial for elevating the SCFA-producing bacteria and play anti-inflammatory roles.^[67]^ For tryptophan metabolites, which are AhR ligands, a clinical trial is being performed to examine the effect of 5-hydroxytryptophan on IBD patients. Another study showed that patients with IBD treated with ursodeoxy cholic acid (UDCA) had a reduced risk of colorectal dysplasia.^[68]^

### Fecal microbiota transplantation

Fecal microbiota transplantation (FMT) is another potential therapy for IBD. It is an excellent way to reshape the composition of gut microbiota by transferring an infusion of a fecal suspension with the help of endoscopy, capsule or bacterial mixture. Now it has been administered to a very limited number of patients with IBD, and their responses were highly variable. Some clinical studies demonstrated that FMT induced remission of ulcerative colitis in approximately 30% of patients.^[69]^ In responders, both the gut microbiota composition and metabolomic profiles largely moved towards those of the donor after FMT. Consistently, butyrate levels were increased and acetate levels were decreased. FMT may have direct beneficial effects by increasing beneficial metabolites and bacteria, or through activating Toll-like receptors in macrophages through microorganism-associated molecular patterns.^[37]^

## Conclusions

Although more studies are also needed to determine mechanisms underlying intestinal inflammation, macrophages homeostasis appears to be crucial for the occurrence, progression, and resolution of IBD. Furthermore, as a key environmental cue, the microbiota regulates macrophage phenotypes and functions. Based on these findings, therapeutics targeting microbiotamacrophages interactions represent an appealing strategy for restoring tissue homeostasis. However, the effects of gut microbiota exerted on macrophages are also likely be observed in many other cell types, thus identifying the factors and mechanisms that control gut microbiotamacrophage interplay in the intestine could be used for drug discovery.
